# Novel EM based ML Kalman estimation framework for superresolution of stochastic three-states microtubule signal

**DOI:** 10.1186/s12918-018-0631-5

**Published:** 2018-11-22

**Authors:** Vineetha Menon, Shantia Yarahmadian, Vahid Rezania

**Affiliations:** 1 Department of Computer Science, University of Alabama in Huntsville, Huntsville, AL USA; 2 Department of Mathematics and Statistics, Mississippi State University, Starkville, MS USA; 3 Department of Physical Sciences, Macewan University, Edmonton, Canada

**Keywords:** Superresolution, Kalman filtering, Expectation Maximization, Wavelets, Principal component analysis, Mutual information, Missing data

## Abstract

**Background:**

Recent research has found that abnormal functioning of Microtubules (MTs) could be linked to fatal diseases such as Alzheimer’s. Hence, there is an imminent need to understand the implications of MTs for disease- diagnosis. However, studies of cellular processes like MTs are often constrained by physical limitations of their data acquisition systems such as optical microscopes and are vulnerable to either destruction of the specimen or the probe. In addition, study of MTs is challenged with non-uniform sampling of the MT dynamic instability phenomenon relative to its time-lapse observation of the cellular processes. Thus, the above caveats limit the overall period of time that the MT data can be collected, thereby causing limited data availability scenario.

**Results:**

In this work, two novel superresolution frameworks based on Expectation Maximization (EM) based Maximum Likelihood (ML) estimation using Kalman filters (MLK) technique are proposed to address the issues of non-uniform sampling and limited data availability of MT signals. The proposed MLK methods optimizes prediction of missing observations in the MT signal through information extraction using correlation-based patch processing and principal component analysis -based mutual information. Experimental results prove that the proposed MLK-based superresolution methods outperformed nonlinear interpolation and compressed sensing methods.

**Conclusions:**

This work aims to address limited data availability and data/observation loss incurred due to non-uniform sampling of biological signals such as MTs. For this purpose, statistical modelling of stochastic MT signals using EM based ML driven Kalman estimation (MLK) is considered as a fundamental framework for prediction of missing MT observations. It was experimentally validated that the proposed superresolution methods provided superior overall performance, better MT signal estimation using fewer samples, high SNR, low errors, and better MT parameter estimation than other methods.

## Background

Research on Microtubules (MTs) have recently garnered a great level of interest due to its anomalous functioning being associated with the onset of several lethal diseases including Alzheimer’s disease [1], Parkinson’s disease [2], and various forms of cancer [3]. Essentially, MTs are intra-cellular polymers made of tubulin protein dimers [4] which are found in all eukaryotic cells, and play major roles in many intra-cellular activities such as trafficking, mitosis, cell motility [4], and chromosome segregation [5]. Under fixed external conditions, it was first noted that the MTs reached a steady state while randomly switching between polymerization (growth) and depolymerization (shrinking) states [4]. This unique phenomena of MTs randomly switching between the two states was named as “dynamic instability” by Mitchison and Kirschner [4, 6–8]. It was later reported in [9, 10], that the MTs were in fact switching spontaneously between three states, namely, growth (g), pause (p) and shrinkage (s). This three-state dynamic instability model involves eight parameters: six transition rates between the states of growth, pause and shrinkage and growth and shrinkage velocities *v*_*g*_ and *v*_*s*_. The average length and mean lifetime of a MT depends on the threshold quantity of these parameters.

Optical microscopes have been utilized for decades as a mainstay for data collection of various cellular processes, such as MTs. Even after modern technological advances in the area of microscopy like single molecule sensitivity, and frame rates in microseconds, optical microscopes are still vulnerable in either damaging the specimen of interest, or the probe during the course of experiment. Moreover, the probe can only be illuminated for a certain period of time, making data collection an arduous task, and data availability is rendered-sparse with respect to the time scale of intra-cellular processes [11, 12]. Especially, in case of MTs, where its high temporal resolution is imperative to estimate the dynamic instability parameters, understand the MT behavior, and other cellular activities. In particular, estimation of MT dynamic instability parameters is crucial to understand the state of the MT system and study its relationships to the occurrence of fatal diseases like Alzheimer’s. Consequently, it signifies the need for stochastic methods that can analytically overcome the challenges such as scarce data availability, and equipment limitations through better signal reconstruction and estimation techniques.

Many statistical methods for modeling stochastic signals have been suggested in literature such as hidden Markov models (HMMs) for capturing the underlying signal variability [13]. As a variation of traditional HMMs, many wavelet-based HMMs have evolved because of their capability to model real-world non-Gaussian signals [14, 15]. Compressed sensing (CS) [16, 17] and other subspace learning methods [18] have also been proposed to reconstruct signals using fewer samples than the Nyquist rate. In literature, it has also been proposed to use maximum likelihood (ML) technique to estimate parameters of a linear dynamic system from the observed data [19, 20]. Typically, this involves formulation of a time-varying Kalman predictor with a likelihood function to minimize the prediction error. In this case, a convergence assumption is made for sufficiently large observations which transforms the minimization of likelihood function to a nonlinear programming problem [21–23]. As an alternative, it was proposed to use Expectation-Maximization (EM) based maximum likelihood (ML) estimation in Kalman filters with the assumption that the state is now observable. This assumption simplifies the convergence criteria by eliminating the need for a large observation limit and facilitates to solve cases with missing observations [24]. Kalman filters have been proved to be optimal in minimizing mean square error sense, under the assumption that all noise is Gaussian. Therefore, the use of EM- based ML estimation in Kalman filters as a means to study MTs is very relevant, since we want to minimize the residual error between the original MT signal, and the predicted MT signal.

In this paper, the MT signal is modelled as a three-state stochastic random evolution signal on which non-uniform sampling is performed to emulate the data loss in real-world scenario [11, 12]. Our motivation is to improve prediction of the missing time-lapse intra-cellular observations analytically; in an effort to minimize the effect of external dependencies such as spatio-temporal resolution, and experimental equipment precision. For this, we propose two novel methods for superresolution of MT signals based on non-uniform sampling, using EM based ML estimation Kalman filter for better prediction of the interpolated MT signal, which is followed by correlation coefficient (R)- based patch processing (MLK-R) [25] and principal component analysis (PCA)-based mutual information (MI) criterion (MLK-MI) for information extraction to further optimize our final predicted MT signal. We estimate the dynamic instability parameters for three states in MTs using wavelet-based peak detection and compare it with our previous work using CS [16]. Our proposed methods MLK-R and MLK-MI gave superior overall performance compared to all other methods, better MT signal recovery and gave high SNR with low errors validating the efficacy of our approaches.

## Methods

### Non-uniform sampling and interpolation

Consider a stochastic input MT signal ***x***(*n*) with *N* samples. Non-uniform sampling is performed on this MT signal ***x***(*n*) to generate its downsampled low resolution version ***x***_*l*_(*n*). The non-uniform sampling case is specifically considered to emulate the loss of information occurring in the real-world scenario due to hardware limitations of the data acquisition system [11, 12]. Therefore, the assumption is that the only signal available at hand for data analysis is the low resolution MT signal ***x***_*l*_(*n*). To accommodate the missing values due to non-uniform sampling, a nonlinear interpolation scheme such as piecewise linear interpolation is then used on the low resolution MT signal ***x***_*l*_(*n*) to get its corresponding high resolution interpolated version ***x***_*h*_(*n*). From this high resolution interpolated frame we can generate a dictionary of *d* new low resolution frames through interlaced non-uniform sampling, where each frame is derived with a constant shift factor *ε*_*i*_ from the first frame as below:

1 $$ {}{\begin{aligned} \boldsymbol{x}_{li}(n) =\{\boldsymbol{x}(mod(\epsilon_{i},N)),\boldsymbol{x}(mod(d+\epsilon_{i},N)), \boldsymbol{x}(mod(2d+\epsilon_{i},N)),\ldots,\\ \boldsymbol{x}(mod((s-1)d+\epsilon_{i},N))\} \end{aligned}}  $$

where *i*=2,…,*d*+1, and ***x***_*l*1_(*n*)=***x***_*l*_(*n*), *mod*=*modulo* operator. For simplicity, we choose *ε*_*i*_=*i*+1, and *d* is the resolution factor and it also denotes the level of downsampling performed. For e.g. *d*=2 implies that the signal was downsampled by a factor of 2. *s* is the number of samples in each frame, calculated as $ s= \left \lfloor \frac {N}{d}\right \rfloor $. For consistency, nonlinear interpolation using piecewise linear interpolation method is performed on the *d* new low resolution MT signals ***x***_*li*_(*n*) to get their corresponding high resolution MT signals ***y***_*hi*_(*n*).

### Expectation-maximization based maximum likelihood estimation of a stochastic MT system

From the previous section, it is assumed that we are only given the low resolution MT signal and that nonlinear interpolation is used as a basis to provide a first estimate for missing signal values and yield the corresponding high resolution MT signals. Thus, the problem of estimation of missing observations of a stochastic MT signal from few known measurements can be formulated as a *d* series of Maximum Likelihood (ML) parameter estimation for a time-varying linear dynamic MT system from observations at hand ***y***_*hi*_(*n*). Our goal is to achieve the best approximation $\hat {\boldsymbol{x}}_{hi}(n)$ of the unknown input signal (or state vector) ***x***(*n*) from observed ***y***_*hi*_(*n*). Let the set of observations ***Y***={***y***_*h*1_(*n*),***y***_*h*2_(*n*),…,***y***_*hd*+1_(*n*)} be generated by a linear dynamic MT system. Then the time-varying Kalman filter equations describing the system can be formulated as below (using notation ***x***_*n*_=***x***(*n*)):

2 $$ \begin{aligned} & \boldsymbol{x}_{n+1}&= \boldsymbol{F}\boldsymbol{x}_{n}+\boldsymbol{w}_{n}\\ & \boldsymbol{y}_{n}&= \boldsymbol{H}\boldsymbol{x}_{n}+\boldsymbol{v}_{n} \end{aligned}  $$

The ***F*** matrix represents the state at the previous time step *n* to the state at the current step *n*+1 in the absence of process noise. The matrix ***H*** gives the relationship between the state ***x***_*n*_ to the measurement ***y***_*n*_. Both process noise ***w***_*n*_ and measurement noise ***v***_*n*_ are assumed as uncorrelated zero mean white Gaussian noise vectors with covariances:

3 $$ \begin{aligned} \boldsymbol{E}\left\{\boldsymbol{w}_{n}\boldsymbol{w}_{m}^{T} \right\}&=\boldsymbol{Q}\\ \boldsymbol{E}\left\{\boldsymbol{v}_{n}\boldsymbol{v}_{m}^{T}\right\}&=\boldsymbol{R} \end{aligned}  $$

Where *T* denotes transpose of a matrix, ***Q*** represents process noise covariance and ***R*** for measurement noise covariance. In practice, the matrices ***F***, ***H***, ***Q*** and ***R*** are subject to change with each time step or measurement (See [22] for detailed information). It is also assumed that the initial conditions for state ***x***_0_ is Gaussian with a known mean and covariance (*μ*_0_,*Σ*_0_). Theerefore, the ML estimates of the unknown parameters *θ* in ***F***, ***H***, ***Q***, ***R***, can be obtained by minimizing the negative log likelihood or as in [19, 20]:

4 $$ {}{\begin{aligned} \boldsymbol{J}(\boldsymbol{Y},\theta) &= - \boldsymbol{L}(\boldsymbol{Y},\theta)\\ & = \sum\limits_{n=0}^{N} \{ \log |\Sigma_{e_{n}}(\theta)|+ \boldsymbol{e}_{n}^{T}(\theta)\Sigma_{e_{n}}^{-1}(\theta)\boldsymbol{e}_{n}(\theta) +constant \end{aligned}}  $$

Where ***e***_*n*_, $\Sigma _{e_{n}}$ are the prediction error and its covariance, and can be obtained from Kalman filter time and measurement update equations as in [20, 22] below:

Time update:

5 $$ \begin{aligned} \hat{ \boldsymbol{x}}_{n+1|n}&= \boldsymbol{F} \hat{ \boldsymbol{x}}_{n|n}\\ \Sigma_{n+1|n}&= \boldsymbol{F} \Sigma_{n|n}\boldsymbol{F}^{T}+\boldsymbol{Q} \end{aligned}  $$

Measurement update:

6 $$ \begin{aligned} \boldsymbol{e}_{n}&=\boldsymbol{y}_{n}- \boldsymbol{H}\hat{ \boldsymbol{x}}_{n|n-1} \\ \Sigma_{e_{n}}&=\boldsymbol{H}\Sigma_{n|n-1}\boldsymbol{H}^{T}+ \boldsymbol{R} \\ \hat{ \boldsymbol{x}}_{n|n}&= \hat{ \boldsymbol{x}}_{n|n-1}+ \boldsymbol{K}_{n}\boldsymbol{e}_{n}\\ \boldsymbol{K}_{n} &= \Sigma_{n|n-1}\boldsymbol{H}^{T}\Sigma_{e_{n}}^{-1}\\ \Sigma_{n|n}&=\Sigma_{n|n-1}-\boldsymbol{K}_{n}\Sigma_{e_{n}}\boldsymbol{K}(n)^{T} \end{aligned}  $$

where ***K*** is the Kalman gain. The function of the measurement update is to adjust the projected estimate by the actual measurement at that time *n*, whereas time update serves the purpose of prediction of the current state estimate $\hat { \boldsymbol {x}}_{n+1|n}$ ahead of time (indicated through future time instance *n*+1). This recursive update is performed with the objective of minimizing (4) with respect to unknown parameter *θ* to estimate the missing observations. In this work, we have used Expectation Maximization (EM) algorithm to find the ML estimates as in [22–24], by the assuming that the state is now observable and can be denoted as ***X***={***x***_*h*_(0),…,***x***_*h*_(*n*)}, hence the problem in (4) can be reformulated as:

7 $$ {}{\begin{aligned} \boldsymbol{J}(\boldsymbol{X},\boldsymbol{Y},\theta) & = - \boldsymbol{L}(\boldsymbol{X},\boldsymbol{Y},\theta)\\ & = \sum\limits_{n=1}^{N} \left\{ \log |\boldsymbol{Q}|+ (\boldsymbol{x}_{n}-\boldsymbol{F}\boldsymbol{x}_{n-1})^{T} \boldsymbol{Q}^{-1} \left(\boldsymbol{x}_{n}-\boldsymbol{F}\boldsymbol{x}_{n-1}\right)\right\}\\ &+ \sum\limits_{n=1}^{N} \left\{ \log |\boldsymbol{R}|+ (\boldsymbol{y}_{n}-\boldsymbol{H}\boldsymbol{x}_{n})^{T} \boldsymbol{R}^{-1} (\boldsymbol{y}_{n}-\boldsymbol{H}\boldsymbol{x}_{n}\right\} +constant \end{aligned}}  $$

Using fixed interval form of Kalman filter (RTS smoother), the new system recursion updates can be computed as in [22]:

Forward Recursions:

8 $$ \begin{aligned} \boldsymbol{e}_{n}&=\boldsymbol{y}_{n}- \boldsymbol{H}\hat{ \boldsymbol{x}}_{n|n-1} \\ \Sigma_{e_{n}}&=\boldsymbol{H}\Sigma_{n|n-1}\boldsymbol{H}^{T}+ \boldsymbol{R} \\ \hat{ \boldsymbol{x}}_{n|n}&= \hat{ \boldsymbol{x}}_{n|n-1}+ \boldsymbol{K}_{n}\boldsymbol{e}_{n}\\ \hat{ \boldsymbol{x}}_{n+1|n}&= \boldsymbol{F}\hat{ \boldsymbol{x}}_{n|n}\\ \boldsymbol{K}_{n}& = \Sigma_{n|n-1}\boldsymbol{H}^{T}\Sigma_{e_{n}}^{-1}\\ \Sigma_{n|n}&=\Sigma_{n|n-1}-\boldsymbol{K}_{n}\Sigma_{e_{n}}\boldsymbol{K}_{n}^{T}\\ \Sigma_{n,n-1|n}&=(\boldsymbol{I}-\boldsymbol{K}_{n}\boldsymbol{H})\boldsymbol{F}\Sigma_{n-1|n-1}\\ \Sigma_{n+1|n}&= \boldsymbol{F} \Sigma_{n|n}\boldsymbol{F}^{T}+\boldsymbol{Q} \end{aligned}  $$

Backward Recursions:

9 $$ {} \begin{aligned} \boldsymbol{A}_{n}&= \Sigma_{n-1|n-1}\boldsymbol{F}_{n-1}^{T}\Sigma_{n|n-1}^{-1} \\ \hat{ \boldsymbol{x}}_{n-1|N}&= \hat{ \boldsymbol{x}}_{n-1|n-1}+ \boldsymbol{A}_{n}\left[\hat{ \boldsymbol{x}}_{n|N}-\hat{ \boldsymbol{x}}_{n|n-1}\right]\\ \Sigma_{n-1|N}&=\Sigma_{n-1|n-1}+\boldsymbol{A}_{n}\left[ \Sigma_{n|N}-\Sigma_{n|n-1}\right]\boldsymbol{A}_{n}^{T}\\ \Sigma_{n,n-1|N}&=\Sigma_{n,n-1|n}+\left[\Sigma_{n|N}-\Sigma_{n|n}\right]\Sigma_{n|n}^{-1}\Sigma_{n,n-1|n} \end{aligned}  $$

Note that the formulation of *d* series estimation of the state vector ***x***_*n*_ is made with the assumption that available data is the observed vector ***y***_*n*_ and it provides *d* unique estimated MT signals $\hat { \boldsymbol {x}}_{hi}(n)$. In each iteration, EM algorithm computes the data-sufficient statistics in recursions (8), (9) and estimates previous model parameters (E-step). New system parameters are obtained from these statistics in the maximization step (M-step).

### Correlation coefficient-based-patch processing

Typically, employing correlation-based approaches are a common practice in superresolution for images. This is because correlation coefficient (R) is highly effective in detection and extraction of identical information present in the low resolution images. Hence, inspired by the proficiency of correlation coefficient in information extraction, we have implemented a novel correlation coefficient (R)- based patch processing technique to find an identical match between similar signal patches (segments) present in the *d*+1 Kalman-predicted MT signals $\hat { \boldsymbol {x}}_{hp}(n)$ and achieve further optimization to yield an enhanced final prediction for MT signal. This is accomplished by using a sliding window *w*=4(*w*=1×4) across the pairwise MT signals. The pairwise R comparisons ensures that the inconsistencies such as artificial low/high frequencies introduced during signal sampling or processing are removed, and maximum information content is extracted from the aliased signals. In order to define a patch as similar or identical the *R* threshold parameter is chosen as *R*>0.90. The MT signal patches that satisfy this condition as in (10) are then included in the final predicted signal $\hat {\boldsymbol {x}}_{f}(n)$. An error minimization condition is also imposed such that the selected patch must not only satisfy *R*>0.90, but also minimize the error between the signals as below:

10 $$ {} \hat{\boldsymbol{x}}_{f}(n)=\hat{ \boldsymbol{x}}_{hp}(n) \iff \left(\boldsymbol{R}(\hat{ \boldsymbol{x}}_{hp}, \hat{ \boldsymbol{x}}_{hq}\right)>0.90) \wedge \left(\mathop{\min_{p\neq q}} {|\hat{\boldsymbol{x}}_{hp}-\hat{ \boldsymbol{x}}_{hq}}|\right)  $$

where *p*, *q*=1,2,…,*d*. For *d* Kalman-predicted MT signals $\hat { \boldsymbol {x}}_{hp}(n)$, we will need ^*d*^*C*_2_ signal comparisons to be made. All missing values in the final MT signal $ \hat {\boldsymbol {x}}_{f}(n)$ (for patches < threshold *R*) are computed by taking the mean of the *d* signals values available for that particular time instance. This step is done as a tradeoff to minimize the prediction error for missing values in the original low resolution MT signal. The final *MLK*−*R*-patch processed MT signal $ \hat {\boldsymbol {x}}_{f}(n)$ had better SNR and lower errors than Kalman-predicted (MLK) ($\hat { \boldsymbol {x}}_{hi}(n)$) and interpolated (NL-I) (***y***_*hi*_(*n*)) signals. This final reconstructed MT signal $\hat {\boldsymbol {x}}_{f}(n)$ was used for estimation of the three-state MT dynamic instability parameters in the wavelet domain using peak detection as in our prior works [16, 26].

### Principal component analysis-based mutual information criterion

Principal component analysis (PCA) is a widely used unsupervised learning technique for information extraction and dimensionality reduction of data in multi-disciplinary applications. The principle behind PCA is that it captures the directions of maximum variance (information) in the data, where these directions of maximum variance represent the principal components or eigenvectors of the data [27]. The significance of PCA includes: 1) the principal components are uncorrelated, thus they provide elimination of redundancy in the data. 2) Most of the important information is usually compacted in the first few eigenvectors, thereby providing data compaction and effective dimensionality reduction of data. In this work, we apply PCA to extract information present in the individual *d*+1 MLK- predicted MT signals. Given a data ***x***, it can be described as a linear combination of its eigenvectors using PCA as:

11 $$ \mathbf{A} =\mathbf{x} \mathbf{B}  $$

where **A** denotes the new principal component scores, **B** gives weight vectors such that the linear combination of **xB** maximizes the variance contained in **A** and data **x** can be recovered as:

12 $$ \mathbf{x} =\mathbf{B}^{-1} \mathbf{A}  $$

Mutual information (MI) is a well-known information extraction criterion that measures the similarity of information content or mutual dependence between two random variables. It was first conceptualized by Shannon [28], since then it been widely adopted in a variety of applications, especially in the area of biology [29, 30]. The mutual information *I* between two discrete random variables *X* and *Y* can be described as in [29]:

13 $$  I(X,Y)= \sum\limits_{x \in X} \sum\limits_{y \in Y} p_{X,Y}(x, y) \log \frac{p_{X,Y}(x, y)}{p_{X}(x)p_{Y}(y)}  $$

where *p*_*X*,*Y*_(*x*,*y*) stands for the joint probability density function of *X* and *Y*, and *p*_*X*_(*x*) and *p*_*Y*_(*y*) denote the marginal probability density functions of *X* and *Y* respectively. Intuitively, if random variables *X* and *Y* are independent, then their MI *I*(*X*,*Y*)=0, whereas if they have perfect dependence then their MI tends to infinity i.e., *I*(*X*,*Y*)→*∞*. As defined by Shannon in [28], mutual information *I*(*X*,*Y*) can also be defined in terms of entropy as [28]:

14 $$ I(X,Y) = H(X)+H(Y) H(X,Y)  $$

Where *H*(*X*) denotes entropy of random variable *X*, *H*(*Y*) represents entropy of random variable *Y* and *H*(*X*,*Y*) describes the joint entropy of random variables *X* and *Y*. The above definition for MI can be interpreted in terms of entropy and probability density functions of the corresponding random variables by substituting Eqs. (13) in (16) to yield:

15 $$ I(X,Y) = H(p_{X})+H(p_{Y}) H(p_{X,Y})  $$

**Fig. 1:**
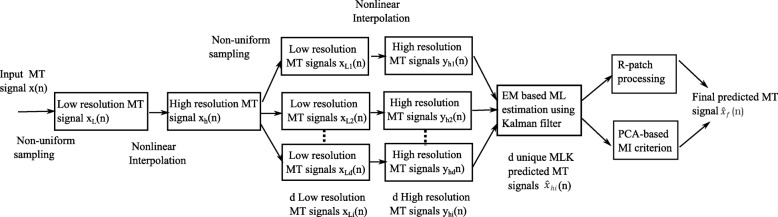
Schematic representation of proposed superresolution framework for MT signal prediction

Thus, in this work, we apply MI to determine the information dependence between the principal components of the *d*+1 predicted MT signals. Greater MI signifies more common information content between the signals. Since we are trying to refine our estimation of the missing values in the MT signal, it is pertinent to retain the common information in the MT predicted signal. Lesser MI could imply a bad signal prediction and therefore should be excluded from analysis to improve final MT signal prediction $ \hat {\boldsymbol {x}}_{f}(n)$. The first two unique principal components *p* corresponding to the MI pairs that give us the maximum MI can be found as below:

16 $$ p=arg \: unique\{ sort_{descend} \{I(x_{ai},x_{aj}) \}\} \\  $$

where *i*,*j*∈{1,*d*+1} and *I*(*x*_*ai*_,*x*_*aj*_) denotes the MI between pairs of principal components *ai* and *aj* of data **x**. The average of *p* principal components selected from our PCA-MI criterion (MLK-MI) is then computed to reconstruct our final MT signal $ \hat {\boldsymbol {x}}_{f}(n)$ using (12). Figure 1 illustrates the block diagram of the proposed superresolution framework for MT signal prediction of missing observations.

### Wavelet transforms

Wavelet transforms are commonly used in a wide variety of biomedical applications like for compression of EEG and ECG signals. Wavelets are specially preferred for modelling stochastic signals due to its attractive benefits such as sparsity, compression, inherent denoising and simultaneous time-frequency resolution of signals. Given a 1D-discrete-time signal $ \hat {\boldsymbol {x}}_{f}(n) $, it can be represented using translations of basis mother wavelet function *ψ*(*n*) and a lowpass scaling function *ϕ*(*n*) in the time and frequency domain as in ([15, 31]) by:

17 $$  \begin{aligned} & \hat{\boldsymbol{x}}_{f}(n) =\sum\limits_{k} c_{j_{0}}(k)\phi_{j_{0},k}(n)+\sum\limits_{j=j_{0}}\sum\limits_{k} d_{j}(k)\psi_{j, k}(n) \end{aligned}  $$

where $ \hat {\boldsymbol {x}}_{f}(n) \in L_{2} (R)$, *k*,*j*∈*Z*. *k* stands for time (translation) and *j* represents the frequency (scale) respectively [31] and the basis functions are:

18 $$  \begin{aligned} \psi_{j,k} (n)&= 2^{\frac{j}{2}}\psi(2^{j} n-k) \\ \phi_{j_{0},k} (n)&= 2^{\frac{j_{0}}{2}}\phi(2^{j_{0}} n-k) \end{aligned}  $$

where the first term in (17) corresponds to the coarse resolution while the second term represents the detail (or wavelet) resolution of the signal. $c_{j_{0}}(k)$ and *d*_*j*_(*k*) are the corresponding approximation (or scale) and detail (or wavelet) coefficients at scale *j*, respectively. These coefficients can be calculated as below [31]:

19 $$  \begin{aligned} c_{j_{0}}(k)&= \langle \hat{\boldsymbol{x}}_{f}(n),\phi_{j_{0},k}(n) \rangle \\ d_{j}(k)&= \langle \hat{\boldsymbol{x}}_{f}(n),\psi_{j,k}(n) \rangle \end{aligned}  $$

Note that *j*_0_ is an arbitrary starting scale. In this paper, the maximum scale *j* also represents the wavelet decomposition levels. We later perform peak detection of MTs in the wavelet domain using the energy packing density (EPE) criterion [32] to estimate the three-state MT parameters. In particular, simultaneous time-frequency resolution is the key wavelet property that will be used for detection of the three-transition states in MTs.

### Trichotomous Markov Noise-based three-states random evolution model

Consider the final predicted MT signal $\hat {\boldsymbol {x}}_{f}(n)$ that is undergoing the dynamic instability phenomena by randomly switching between the three transition states of growth (g), pause (p) and shrinkage (s). We formulate a three-states random evolution model for the study of dynamic instability process in MTs using Trichotomous Markov Noise (TMN), which is a three-state stochastic process. Hence, the three-states random evolution model for the dynamic instability of MTs has eight states-space parameters, including six transition rates between states of growth, pause and shrinkage denoted by *f*_*ij*_, where *i*,*j*∈{*g*,*p*,*s*} and two states of growth and shrinkage rates, represented by *v*_*g*_ and *v*_*s*_ [16]. The mean length and lifetime of a MT in the three-states random evolution model depends on the threshold quantities *f*_*gs*_, *f*_*sg*_, *v*_*g*_, and *v*_*s*_ in the formula for *V* in (20) [33]. If the quantity, *V* is positive, the MTs tend to shrink more than they grow, and the MTs will have a finite mean length and mean lifetime. Otherwise, on average, they tend to grow forever. The derivation of equations for three-states random evolution model for MTs are provided in [16]:

20 $$  \begin{aligned} F_{g}&=f_{gp}+f_{gs},\\ F_{p}&=f_{pg}+f_{ps}\\ F_{s}&=f_{sp}+f_{sg}\\ F&=F_{s}+F_{p}+F_{g}\\ F_{gp}&=f_{gs}f_{pg}+f_{gs}f_{ps}+f_{gp}f_{ps}\\ F_{sp}&=f_{sg}f_{pg}+f_{sg}f_{ps}+f_{sp}f_{pg}\\ F_{sg}&=f_{sp}f_{gs}+f_{sp}f_{gp}+f_{sg}f_{gp}\\ \Omega&=F_{sp}+F_{pg}+F_{sg}\\ V&=\frac{v_{g}{F}_{sp}-v_{s}{F}_{pg}}{\Omega}\\ L&=\frac{v_{s}v_{g}}{v_{s}\frac{F_{gp}}{F_{p}}-v_{g}\frac{F_{ps}}{F_{p}}} \end{aligned}  $$

Where *V* gives the equilibrium point of the system and *L* denotes the average length of the MT signal.

## Results and discussion

In this section, we experimentally validate the effectiveness of our proposed methods for superresolution of MT signals. The MT data used in this work are results of experiments on MTs (composed of purified *αβ*_*II*_ isotopes from bovine brain tubulin) performed by O. Azarenko, L. Wilson and M.A Jordan at the University of California, Santa Barbara. Tubulin proteins were first purified from the bovine brain and then seeded to polymerize at 37°*C*. The growth and shrinkage dynamics of individual purified MTs were then recorded at their plus ends using the differential interference contrast video microscopy. Data points representing MT lengths were collected at 2−6*s* intervals. MT lengths were analyzed using the Real Time Measurement program. Growth and shrinkage rates were calculated by least-squares regression analysis of the data points. Growth and shrinkage thresholds are set to an increase in length by 0.2*μm* at a rate of 0.15*μm*/*min* and a decrease in length by 0.2*μm* at a rate of 0.3*μm*/*min*, respectively. Any length changes equal to or less than 0.2*μm* over the duration of six data points were considered attenuation phases (phases in which length changes were below the resolution of the microscope). It should be noted that the experimental detection limit for length changes corresponds to about 400−800 tubulin dimers. The supplied data, however, was in the form of a hard copy graphs, that they were scanned and then digitized using the software “DigitizeIt” (http://www.digitizeit.de/) [34].

**Fig. 2:**
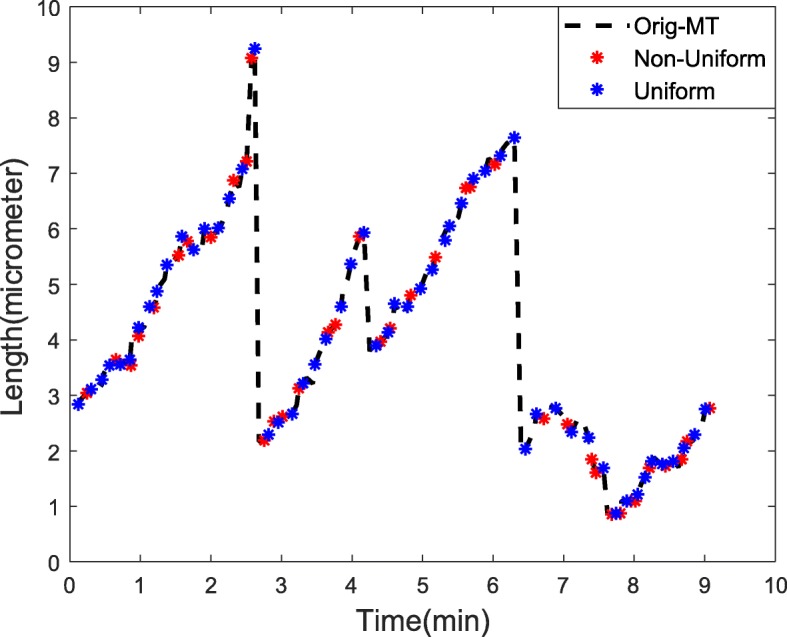
Non-uniform sampling of MT Signal

The MT data used in this study is ABII (*αβ*_*II*_) data and the number of samples present in this original MT signal was *N*=165. In this study, non-uniform sampling was performed on the original MT signal by a factor of *d*=3 to emulate the inadequate MT data collection limitation in physical systems. Figure 2 shows the original MT signal. The *red* points indicate the non-uniformly chosen samples, while *blue* points indicate the samples chosen through uniform sampling by a factor *d*=3. Note that the *blue* points were more evenly distributed, unlike *red* points that tend to be clustered around certain areas indicating a non-uniform distribution/sampling across the MT signal.

In this paper, we introduce two novel methods for enhanced MT signal prediction in the new superresolution framework for MTs. Both the proposed methods have non-uniform sampling performed on the MT signals to emulate real-world data loss scenario. This is followed by EM-based ML Kalman prediction (MLK) of the MT signals as a basis framework for superresolution. The first proposed enhanced prediction method involves using an image processing-inspired correlation (R)-based-patch processing (MLK-R) method to further optimize the final MT predicted signal by extraction of information through identification of similar patches from the dictionary of MLK-predicted MT signals. Second method involves PCA based MI criterion (MLK-MI) method that performs extraction of similar information present in principle components of the MLK-predicted MT signals using MI criterion for elimination of data redundancy and further enhancement of the predicted MT signal. Comparison analysis of the proposed superresolution methods is performed with respect to two other methods, namely, non-uniform sampling of MT signal followed by nonlinear (piecewise linear) interpolation technique (NL-I) and our previous work using compressed sensing (CS) to reconstruct MT signals with fewer samples than the Nyquist rate [16]. Since, the non-uniformly sampled low resolution MT signal was sampled at a factor of *d*=3, this implies that it has $s= \left \lfloor \frac {N}{d}\right \rfloor = \left \lfloor \frac {165}{3}\right \rfloor =55$ samples. This is equivalent to CS subrate of $ \frac {s}{N}= \frac {55}{165}= 0.33$. Therefore, we included results of CS rate =0.3 (CS-0.3) from our prior work [16] for comparison.

**Fig. 3:**
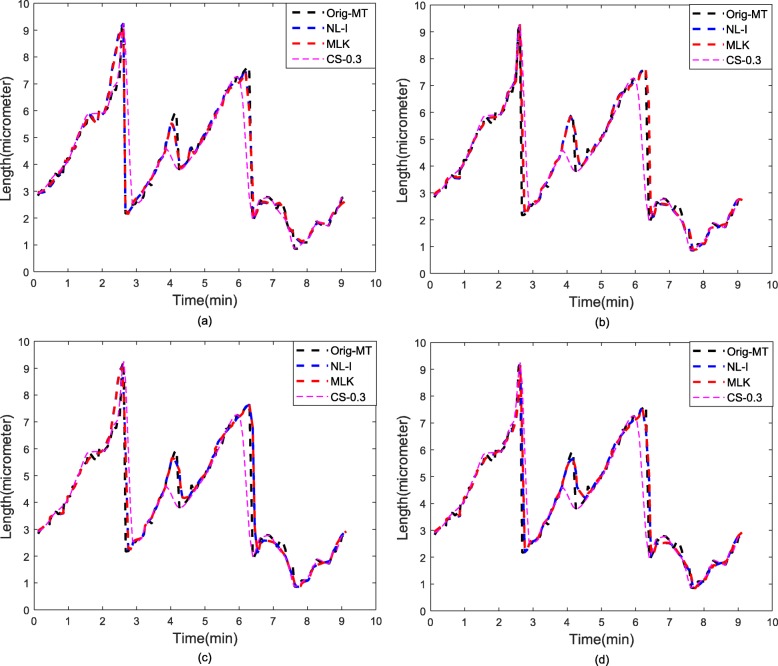
Comparison of EM based ML-Kalman (MLK) predicted MT signals with other methods. **a** MLK-predicted signal d=1 **b** MLK-predicted signal d=2 **c** MLK-predicted signal d=3 **d** MLK-predicted signal d+1=4

**Fig. 4:**
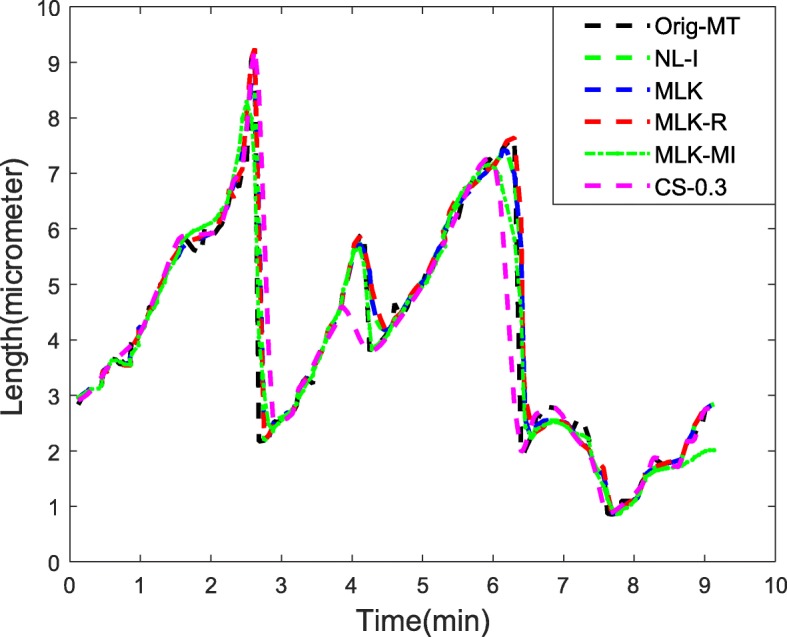
Comparison of our proposed superresolution methods for MT signals with other methods

As discussed under Methods section, after non-uniform sampling is performed to yield the resultant low resolution MT signal, its corresponding high resolution MT signal is then generated using nonlinear interpolation techniques like piecewise interpolation in our case. An interlaced non-uniform sampling of this resultant high resolution interpolated MT signal is carried out to generate *d* -low resolution MT signals and their corresponding high resolution MT signal versions ***y***_*hi*_(*n*) this is denoted as the NL-I method. Subsequently, EM based ML estimation using Kalman filters is applied to these high resolution MT signals to achieve a better prediction of the missing MT values (MLK method). All the methods were followed by peak detection in wavelet domain to estimate the three-state dynamic instability parameters for MTs. Figures 3 and 4 illustrates the final predicted MT signals across all methods, where Fig. 4 shows the average of *d*+1 predicted MT signals for both NL-I and MLK methods. From Figs. 3-4, it can be inferred that our proposed methods MLK-R and MLK-MI had the best final predicted MT signal across all the methods. To quantitatively substantiate the effectiveness of our approach, we compute the signal-to-noise-ratio (SNR) and root mean square error (RMSE) statistics of the predicted MT signals from all methods in Table 1. Again, our proposed superresolution methods MLK-R and MLK-MI had the highest SNR and lowest RMSE of all other methods. In particular, MLK-R outperformed all other methods both qualitatively and quantitatively.

**Table 1:** Comparison of SNR and RMSE for all methods

MT Parameters	MLK-R	MLK-MI	NL-I	CS-0.3
*SNR* (*indB*)	**15.88**	12.71	12.09	4.78
*RMSE*	**0.42**	0.44	0.49	1.10

Significance as referred in main text is that the bold text represents highest / best performance values

The main difference between our proposed superresolution framework in this study and CS method from previous work [16] is that in the former case we assume that only the low resolution MT signal is available to us and using statistical modelling we try to predict and estimate the missing values in the original MT signal with minimum error possible. Whereas in the latter case, we assume that we have already acquired the original MT signal and want to compress it at the sensor side for storage or transmission purposes such that at the receiver side both the compressed signal and the transformation basis is known. The main reason for wide acceptance of CS is that we can preserve the signal details using a random measurement matrix as Gaussian, thereby eliminating the need for complex processing and transmission of transformation basis. But CS makes no effort in learning the underlying signal characteristics which can be critical in understanding biological processes as in case of MTs. On the other hand, statistical modeling methods like EM based ML estimation based Kalman prediction learns the signal structure and hence provides better signal prediction. This is demonstrated through Fig. 3 and Table 1, wherein MLK-R and MLK-MI methods had higher SNR, lower RMSE and performed better than the CS methods, due to the data-learning and information extraction processes involved. Thus, both our current work and previous work [16] makes an effort to analytically address various facets of the data acquisition problem in MTs and biomedical signals in general, such as low sample availability scenario, spatio-temporal resolution and compression of biomedical signals.

In particular, statistical modelling of MTs as a stochastic signal with the problem of recovering the original signal through fewer samples, while assuming all noise to be Gaussian makes Kalman prediction an obvious choice given its optimality in the mean squared error sense. Kalman filtering is also ideal for real-time signal processing, where the future samples can be predicted in real-time from the adaptive feedback of statistics computed from the current samples. A good prediction of MT signal is quintessential for better estimation of dynamic instability parameters of three-state MTs, understand the MT behavior and state of the MT system. Thus, our MLK-R and MLK-MI method has shown to provide better prediction and estimation of the unknown parameters in the MT linear dynamic system. In addition, wavelet domain MT signal processing is done to exploit benefits such as sparsity, inherent denoising and most importantly simultaneous time-frequency resolution which is very pertinent for good peak/ MT transition state detection. For consistency with our prior work [16], we have used Daubechies D2 (db2) as mother wavelet with 8 levels of signal decomposition; it also performs experimentally better than other mother wavelet families for our work. Wavelet transform is performed on the final predicted MT signal to get its corresponding sparse wavelet coefficients. Wavelet domain peak detection is then employed to detect the peaks indicating the transition points in the MT signal between the growth (g), pause (p) and shrinkage (s) states. This transition information is used to determine when and where the MTs are switching between the three states. This is where the simultaneous time-frequency resolution of wavelets plays a crucial role. Since, we want to detect switching instants (or time), implies we are looking for narrower time bins for better time resolution, which corresponds to the lowest level significant wavelet coefficients (**swc**). As in our prior work [16, 26], energy packing efficiency (EPE) [32] is used to determine the number of significant wavelet coefficients/peaks that need to be chosen. The EPE steps for peak detection of MTs are as follows:

- Step 1: Sort the lowest level wavelet coefficients in descending order (i.e. larger significant wavelet coefficients are most likely peaks).
- Step 2: Compute the total energy present in the wavelet coefficients (**swc**) in the lowest level. Where the total energy is calculated by:
  21 $$ E_{TOT} = \sum{\mathbf{swc}^{2}}   $$
- Step 3: Fix a desired threshold to indicate the percentage of significant wavelet coefficients to be retained. In our case, we retain values such that 85% of total energy of the coefficients in the lowest level is preserved. That is:
  22 $$ E_{TH} \geq 0.85*E_{TOT}   $$
- Step 4: Compute the total energy of the sorted wavelet coefficients (*E*_*TH*_) as in (21), until the threshold condition in (22) is met.

**Fig. 5:**
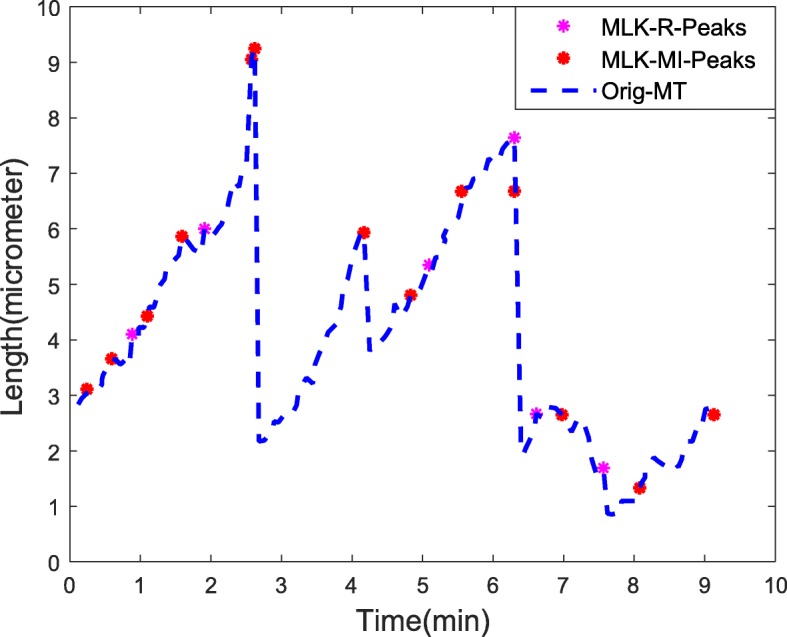
Wavelet domain peak detection of the MLK-predicted MT signal

The value of EPE threshold parameter plays a vital in detection of peaks and three-transition states in MTs. Higher the threshold parameter, lower the number of significant wavelet coefficients selected, thereby fewer peaks/ MT transition states are detected (implying loss of information, if threshold parameter is too high). Lower the threshold, higher the number of false peaks detected, thus causing an over-prediction of the MT transition states. Therefore, the number of significant wavelet coefficients retained is sensitive to threshold chosen, which in turn dictates the number of potential peaks or MT transition states detected. Figure 5 shows the peaks detected in predicted MT signal using wavelet domain EPE method. The information derived from peak detection is then used to encode our final predicted signal to its nearest peak to obtain a TMN modelled three-state MT signal. The resultant encoded three-state MT signal is used to compute MT parameters like transition frequencies, transition velocities and average MT lengths as tabulated in Tables 2 and 3. The error estimates of MT parameters for all methods are given in Table 4. From Tables 2 and 4, we substantiate that overall MLK-R and MLK-MI had the best performance compared to other methods. Specially, our proposed method MLK-R demonstrated superior overall performance, higher SNR, lower RMSE, better MT signal prediction and parameter estimation with lowest errors from fewer samples compared to interpolation and CS methods.

**Table 2:** Comparison of the original and estimated transition frequency MT parameters for all methods

MT Parameters	OrigMT	MLK-R	MLK-MI	NL-I	CS-0.3
*f*_*sg*_	**0.22**	**0.22**	0	**0.22**	**0.22**
*f*_*gs*_	**0.22**	0.44	**0.22**	**0.22**	**0.22**
*f*_*sp*_	**8.76**	**8.76**	5.47	9.53	12.04
*f*_*ps*_	**0.11**	0	0	**0.11**	0
*f*_*gp*_	**0.11**	0	0.22	0	0
*f*_*pg*_	**8.54**	**8.54**	11.59	7.88	5.48

Significance as referred in main text is that the bold text represents highest / best performance values

**Table 3:** Comparison of the original and estimated velocity and length MT parameters for all methods

MT Parameters	OrigMT	MLK-R	MLK-MI	NL-I	CS-0.3
*v*_*s*_	**45.17**	**45.17**	42.16	72.27	63.24
*v*_*g*_	**40.65**	**42.16**	40.11	36.14	21.68
*avg* *L*	**5.24**	5.31	**5.27**	7.87	5.44

Significance as referred in main text is that the bold text represents highest / best performance values

**Table 4:** Comparison of the estimated MT error parameters for all methods

MT Parameters	MLK-R	MLK-MI	NL-I	CS-0.3
*f*_*sg*_	0	0.22	0	0
*error*				
*f*_*gs*_	-0.22	0	0	0
*error*				
*f*_*sp*_	0	-3.28	-0.77	-3.28
*error*				
*f*_*ps*_	0.11	0.11	0	0.11
*error*				
*f*_*gp*_	0.11	0.11	0.11	0.11
*error*				
*f*_*pg*_	0	3.05	0.66	3.07
*error*				
*v*_*s*_	-2.00	-3.00	-4.00	-3.00
*error*				
*v*_*g*_	2.66	3.00	3.00	3.00
*error*				

## Conclusion

In this paper, we propose two novel frameworks for superresolution of MT signals to address the limited data availability scenario and emulate the data loss due to non-uniform sampling of biological/natural signals that we often encounter in the real-world. This work exploits the stochastic nature of MT signals through statistical modelling using EM based ML driven Kalman estimation (MLK) for better prediction of the non-uniformly sampled MT signal as a basic superresolution framework. The MLK-predicted MT signals were further optimized through information extraction using correlation (R)-based-patch processing (MLK-R) and PCA-based mutual information criterion (MLK-MI) methods. We perform comparison analysis of our proposed methods MLK-R and MLK-MI with respect to nonlinear interpolation (NL-I) and CS methods. It was experimentally found that both the proposed methods MLK-R and MLK-MI achieved the best overall performance for MT signal estimation. Specifically, MLK-R outperformed all the methods, and had better reconstruction performance using fewer samples, gave high SNR, low errors, as well as better MT parameter estimation than other compared methods. This work aims to demonstrate the effectiveness and significance of statistical modelling and data learning in MTs, and biomedical paradigm. Our goal is to provide an analytical solution to overcome the equipment/hardware fallacies that might occur during the signal acquisition process.
